# Applied Hepatic Bioengineering: Modeling the Human Liver Using Organoid and Liver-on-a-Chip Technologies

**DOI:** 10.3389/fbioe.2022.845360

**Published:** 2022-02-14

**Authors:** Kayque Alves Telles-Silva, Lara Pacheco, Sabrina Komatsu, Fernanda Chianca, Luiz Carlos Caires-Júnior, Bruno Henrique Silva Araujo, Ernesto Goulart, Mayana Zatz

**Affiliations:** ^1^ Human Genome and Stem-Cell Research Center (HUG-CEL), Institute of Biosciences, University of Sao Paulo (USP), Sao Paulo, Brazil; ^2^ Lieber Institute for Brain Development, School of Medicine, Johns Hopkins University, Baltimore, MD, United States

**Keywords:** liver, hepatocyte, 2D cell culture, liver organoid, liver-on-a-chip, drug screening, liver spheroid

## Abstract

The liver is the most important metabolic hub of endo and xenobiotic compounds. Pre-clinical studies using rodents to evaluate the toxicity of new drugs and cosmetics may produce inconclusive results for predicting clinical outcomes in humans, moreover being banned in the European Union. Human liver modeling using primary hepatocytes presents low reproducibility due to batch-to-batch variability, while iPSC-derived hepatocytes in monolayer cultures (2D) show reduced cellular functionality. Here we review the current status of the two most robust *in vitro* approaches in improving hepatocyte phenotype and metabolism while mimicking the hepatic physiological microenvironment: organoids and liver-on-chip. Both technologies are reviewed in design and manufacturing techniques, following cellular composition and functionality. Furthermore, drug screening and liver diseases modeling efficiencies are summarized. Finally, organoid and liver-on-chip technologies are compared regarding advantages and limitations, aiming to guide the selection of appropriate models for translational research and the development of such technologies.

## Introduction

The liver is the largest solid organ in the human body with a highlighted capacity of regeneration, acting distinctively as a regulator of blood sugar and ammonia levels, a synthetic hub of hormones, plasma proteins (e.g., albumin, fibrinogen and transferrin), and bile, a storage center of iron and vitamins as well as a main metabolizer of endogenous and exogenous compounds (Boeri et al., 2019).

Two million deaths per year worldwide are associated with liver diseases, which are triggered by environmental cues (Ehrlich et al., 2019). The main source of acute and chronic liver failure is alcohol consumption, leading to alcoholic liver disease (ALD) or cirrhosis (Moradi et al., 2020). Non-alcoholic fatty liver (NAFL) and non-alcoholic steatohepatitis (NASH) (Soret et al., 2021) can evolve to hepatocellular carcinoma (HCC) and liver cirrhosis, manifested as a result of metabolic syndromes and obesity, which encompass up to one-third of the western population (Younossi et al., 2019). Hepatitis B and C are the major causes of viral liver disease, affecting over 71 million people in 2019 as estimated by the WHO, higher numbers than HIV-related cases (Kulkeaw and Pengsart, 2021).

The development of new drugs by the pharmaceutical industry takes approximately a decade, with estimated one in 5000 drug candidates completing the journey. This inefficiency is due to identification of cardiac toxicity and, most frequently, drug-induced liver injury (DILI) (Skardal et al., 2020). Market withdrawal of approved drugs represents a financial cost of approximately U$ 2 Bi annually for the pharmaceutical industry and a massive health care cost (Grattagliano et al., 2002). In the United States, 5.3% of hospitalizations are related to adverse drug reactions (Jang et al., 2019).

**FIGURE 1:**
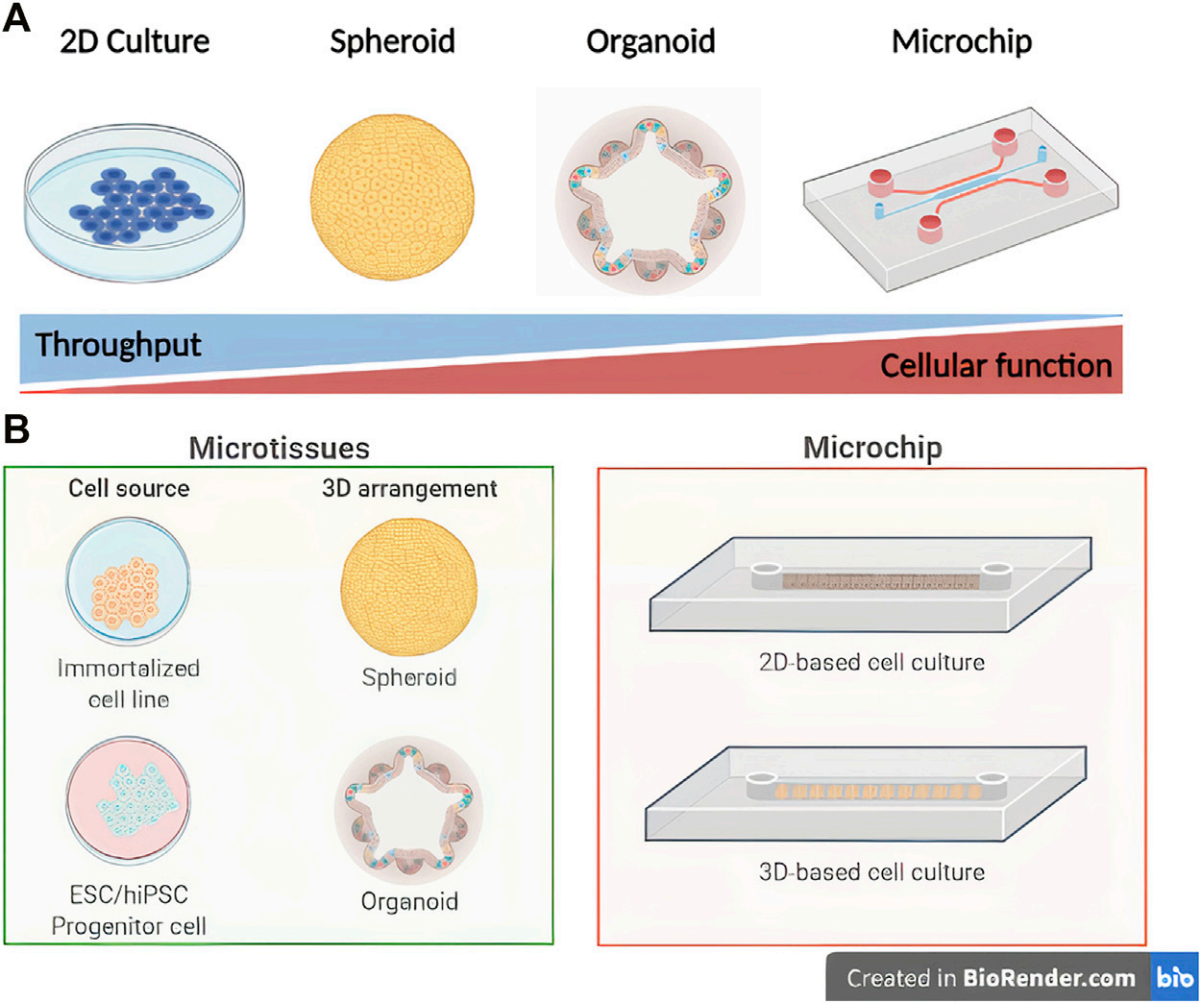
Schematic representation of *in vitro* liver engineering modeling platforms. Comparison of throughput level versus hepatocyte biological function **(A)** and subdivisions of microtissue/microchip techniques **(B)** (Created with BioRender.com).

Despite major advances, modeling human liver physiology is still a bottleneck (Van Midwoud et al., 2011). In this review, we discuss the applications and limitations of current *in vitro* models and how organoid and liver-on-a-chip technologies leverage liver function studies, disease modeling, and new drugs evaluation (Figure 1).

## The Human Liver

**FIGURE 2:**
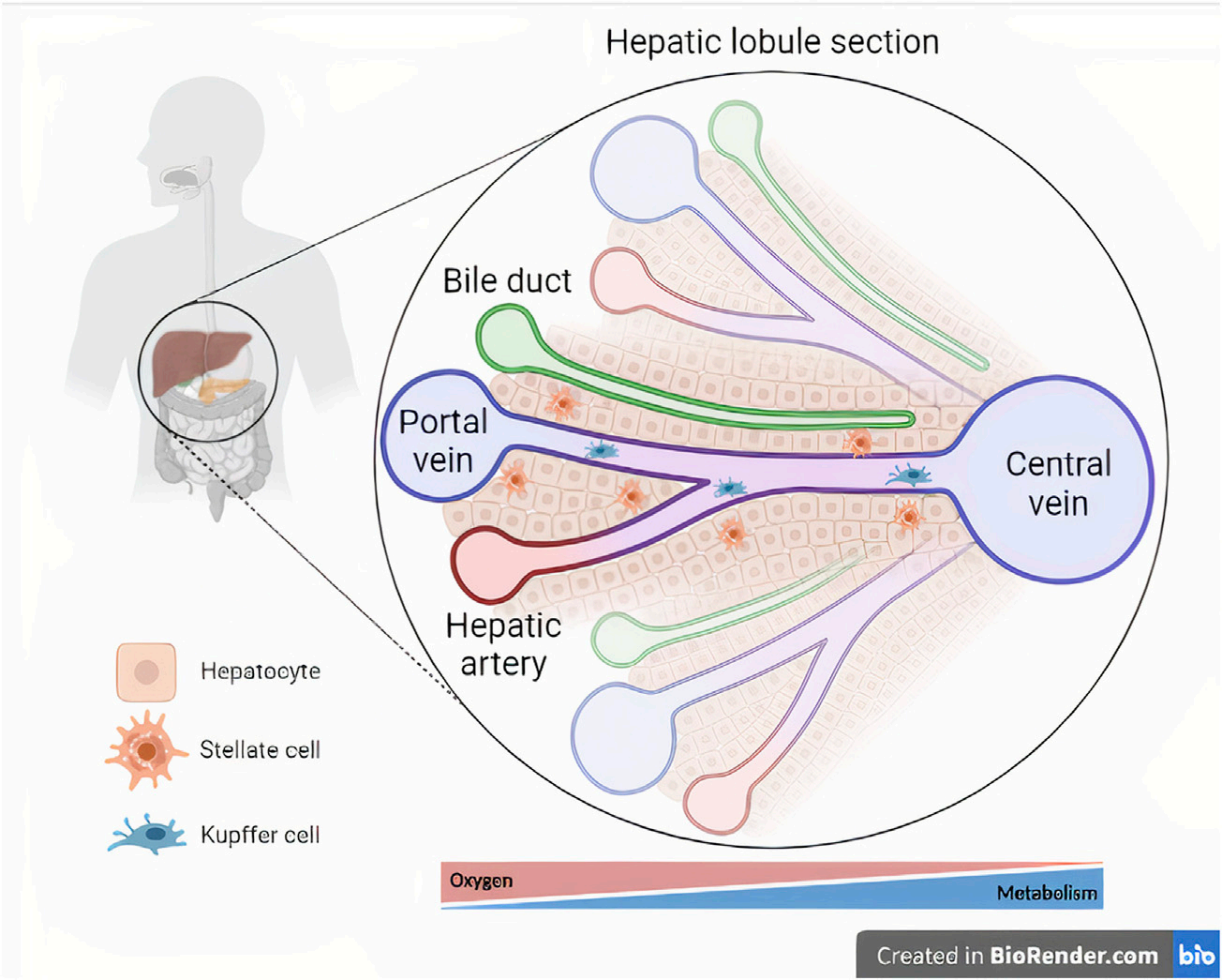
Cellular composition and microarchitecture of the liver. The liver tissue is magnified within a section of a hepatic lobule, representing the central vein and its associated hepatic triads (i.e., bile duct, hepatic artery, and portal vein). Oxygen- and metabolism-driven zonation is represented below the hepatic lobule section (Created with BioRender.com).

The liver comprises a combination of resident parenchymal cells (hepatocytes) and nonparenchymal cells (NPCs), such as cholangiocytes (i.e., biliary duct cells), hepatic stellate cells (HSCs), Kupffer cells, and liver sinusoidal endothelial cells (LSECs) (Guo et al., 2011) (Figure 2). As the main component of the liver, representing 80% of the organ mass (Godoy et al., 2013), hepatocytes are responsible for the metabolism of endogenous and exogenous molecules (Usta et al., 2015) mediated by cytochrome P450 (CYP450) enzymes (Deng et al., 2019). The access to gradients of hormones, nutrients and oxygen pressures results in the metabolic zonation of hepatocytes (Lasli et al., 2019). Another important feature of hepatocytes, as epithelial cells, is polarization, allowing the traffic of compounds from the blood to the bile for excretion (Treyer and Müsch, 2013).

## Current Liver Models

Liver failure modeling and drug screening can be designed *in vivo* or *in vitro*. *In vivo* liver disease models comprise genetic or feeding-induced ALD or NAFLD rodent strains, while preclinical studies use non-rodent animals (Santhekadur et al., 2018). *In vitro* techniques include isolated liver systems, precision-cut liver slices, primary cell culture from liver tissue, cell culture systems derived from stem cells, like 2D and 3D or organoid models, 3D bioprinted organs, and liver-on-a-chip systems (Ehrlich et al., 2019).

Regulatory agencies worldwide require the safety evaluation of new drug candidates for approval. Rodent and non-rodent experimental models are the main preclinical models for promoting drugs to human clinical trials (Olson et al., 2000). However, the European Commission banned the commercialization of cosmetics composed of animal-tested chemicals (EU, 2014) and the FDA released a guideline for reducing, refining and replacing animals in research and industry (Monticello et al., 2017).

Variations in genetics, metabolism and physiology between humans and animal models impair the validity of *in vivo* testing (Martignoni et al., 2006). Liver injury models present low reproducibility and are highly laborious, with clinical trials showing that nearly half the drugs causative of DILI are not identified as damaging by animal models (Olson et al., 2000), while the toxicity evaluation of 150 drugs responsible for adverse effects in humans using dogs and rats presented an accuracy of only 71%, contributing for market withdrawal of approved drugs and clinic drug failure (Monticello et al., 2017). Thus, modeling liver diseases using more accurate human liver models reduces the use of animals, increases testing efficiency, and promotes cost reduction.

## *In vitro* Liver Modeling

Monolayer cell culture represents the most diffused *in vitro* method for liver modeling (Weng et al., 2017). The selection of appropriate cell sources, medium formulation, and oxygen access has been the main barrier in mimicking liver metabolic activity and physiology, impairing the achievement of organotypic liver models, especially regarding its intricate structure and multitasking metabolism (Ziółkowska et al., 2011).

Primary human hepatocytes (PHHs) are the gold standard for cell culture, presenting intrinsic ammonia detoxification, glucose synthesis and phase I and II metabolic activities (Desai et al., 2017), although fast dedifferentiation on dishes and low cell yield hamper long-term studies (Lauschke et al., 2016). Additional widely used cell sources are immortalized (Weltin et al., 2017), transdifferentiated (Ullah et al., 2015), and hiPSC-derived hepatocytes, or hepatocyte-like cells (HLCs) (Yamanaka, 2020). Commercial hepatic cell lines, derived from classic human hepatocellular carcinoma progenitor (HepG2/C3A) or adult (HepaRG) cells, and modern alternatives from expanded primary hepatocytes (e.g., Upcyte hepatocytes and Corning HepatoCells), rapidly expand in culture, but show low CYP450 activity, ALB secretion, and nonfunctional urea cycle (Levy et al., 2015). Hepatocytes derived from mesenchymal stem cells (MSCs) or fibroblasts are also highly proliferative but require ectopic expression of liver-specific transcription factors for differentiation and still undergo dedifferentiation-driven loss of phenotype (Ullah et al., 2015). HLCs achieve an unlimited cell source with low batch-to-batch variability and patient-specific genotype, compatible with personalized medicine perspectives, although presenting fetal liver molecular markers, low CYP450 function, and requires complex cell manipulation (Wang et al., 2017).

The restricted 2D-cultured hepatocytes metabolic functions and cell-cell communication at physiological levels hamper conventional *in vitro* disease modeling and drug screening (Materne et al., 2013). The 2D co-culture of hepatocytes with nonparenchymal cells (NPCs) directs the recapitulation of liver-specific functions through heterotypic cell-cell interactions with cholangiocytes and sinusoidal endothelial cells, extracellular matrix renewal by hepatic stellate cells (HSCs), immune modulation by Kupffer cells, and hepatocyte polarization CYP450-mediated and ALB secretion enhancement (Deng et al., 2019). However, the complexity and cost of co-culture experiments are an important limitation and as the cells are randomly arranged, *in vivo* models are still recommended for liver injury and pharmacokinetic studies (Wirtz et al., 2011). Furthermore, static 2D cultures are unable to supply adequate levels of oxygen for highly-demanding hepatocytes (Nahmias et al., 2006) and medium formulations frequently contain growth factors and hormones in supraphysiological concentrations (Matthew et al., 1996), impairing hepatic zonation (Allen and Bhatia, 2003) while resulting in dysfunctional cell activity and gene expression. Thus, monolayer cell culture is widespread, cheaper, easier to maintain, and suitable as alternatives to animal models for drug safety and screening only of early stage drugs candidates.

Liver organoids, the heterotypic parenchymal and NPC modification of 3D spheroids (Huch and Koo, 2015), are increasingly spreading as a technology that reproduces the liver development natural or synthetic scaffolds resulting on high yield and liver-specific genetic and metabolic accuracy (Goulart et al., 2019). Liver-on-a-chip devices can incorporate liver organoids and other technologies to better recapitulate liver diseases and drug response (Khetani and Bhatia, 2008). As the most promising 3D approaches, liver organoids and liver-on-a-chip recent developments are further detailed in this work.

## Microtissues

Spheroids and organoids, also defined as microtissues, are the main three-dimensional cell culture alternatives developed to resemble hepatic interaction and architecture *in vitro* (Sato et al., 2021). Spheroids represent homo- or heterotypic 3D simple broad-ranging cell aggregates derived from scaffold or droplet-based techniques (Mischiati et al., 2015). Organoids, or mini-organs, are defined as complex organ-specific, self-organizing, and stem- or progenitor-cell derived 3D structures with the capacity to reproduce the physiology of native organs (Huch and Koo, 2015). Liver organoids (LOs) have emerged as an important tool for drug screening, disease and human organogenesis modeling, and personalized medicine (Jin et al., 2021),better recapitulating the liver physiology when compared to animal models or monolayer platforms (Kammerer, 2021).

LO formation involves the self-organization of a defined cellular composition (Zhu et al., 2021). The signaling environment is essential for cellular proliferation and differentiation. Hydrogels, categorized as natural or synthetic, are mainly used to produce LOs (Ye et al., 2019). Natural hydrogels have several advantages such as ECM mimicking and microenvironment maintenance, providing mechanical and biochemical cues to cells. However, there are some components that are variable and not chemically defined, making it difficult to control the consistency and reproducibility of large-scale organoid production (Zhu et al., 2021).

3D hepatic organoids mostly rely on matrigel, a natural hydrogel made of a protein mixture secreted by Engelbreth-Holm-Swarm murine sarcoma cells (Ramli et al., 2020), LO improvement relies on increasing cellular diversity by mimicking the liver compositing *in vitro*, using distinct NPCs (Kammerer, 2021). Ramli et al*.* developed a liver organoid with hepatocytes and cholangiocytes, recapitulating a single contiguous canaliculi network and modeling NAFLD progression (Ramli et al., 2020). Ouchi et al. cultured a liver organoid composed of hiPSC-derived HSCs and Kupffer cells, exhibiting high CYP3A4 activity after 48 h of rifampicin exposure (Ouchi et al., 2019). Inter-organ organoids also demonstrate the importance of multiorgan connections to refine the functions of *in vitro* systems. Koike et al*.* designed hepato-biliary-pancreatic organoids by differentiating PSCs into anterior and posterior gut spheroids and fusing them together in one cell culture well (Koike et al., 2021). Nevertheless, matrigel-based techniques are limited by batch-to-batch variability and xenogenic contaminants, leading to a lack of control of the organoid formation and reproducibility in cell culture experiments (Aisenbrey and Murphy, 2020).

There are several researchers using different types of natural hydrogels to generate liver organoids such as protein-based and polysaccharide hydrogels (Katyal et al., 2020). Among natural biomaterials, ECM hydrogel derived from decellularized tissues have been studied and used to create LO (He et al., 2020). Although natural hydrogels better mimic the ECM, synthetic hydrogels are widely used while having stronger structures, lower complexity, easier controllability, and a tendency to be approved by the FDA (Ye et al., 2019).

As an alternative to natural ECM-based hydrogels, several variations of synthetic hydrogels have been applied to LOs (e.g., PEG) to better mimic specific liver physiological and pathological conditions (Sorrentino et al., 2020). Klotz et a*l.* proposed a hybrid hydrogel based on PEG and gelatin that outperforms matrigel when modeling liver by establishing a liver-specific ECM-mimicking matrix, increasing the activity of CYP450 protein members (Klotz et al., 2019). Sekine et al. developed a chemically defined animal-free origin composition for every LO, promoting standardization and reproducibility of liver organoids (Sekine et al., 2020). Sorrentino et al. modeled the fibrotic liver by establishing a liver organoid using PEG and tuning the stiffness of the synthetic networks to physiological parameters (Sorrentino et al., 2020). Funfak et al*.* studied the cellular microenvironment on differentiation, physiology, and organogenesis of cholangiocytes into functional biliary tubes by constructing a cholangiocyte organoid with PEG-based hydrogel (Funfak et al., 2019). Ye et al. used hydrogels based on polyisocyanopeptides (PIC) for the expansion and differentiation of LOs that presented intracellular concentration of ALB and GLDH (Ye et al., 2020).

## Liver-On-A-Chip

Microfluidic devices emerged to mimic tissue microenvironments at the submillimeter scale (Ehrlich et al., 2019), overcoming the main challenges of static 2D liver modeling through enhanced control over cell culture medium flow, oxygenation, and biomolecule gradient-generation conditions, resembling *in vivo* advantages of immune response, chemotaxis, as well as cell position-mediated activity and differentiation artificially *in vitro* (Eckstrum et al., 2020).

Liver-on-a-chip platforms are modeled to simulate functional hepatic units able to identify hepatotoxic and DILI-inducing drugs, to examine the liver regeneration capacity and physiology, and to model ALDs and NAFLDs (Hou et al., 2020). Liver-on-a-chip manufacturing involves fabrication through biomaterial-based microcontact printing or micropatterning techniques, creating microchambers connected by channels adequate for co-culture and culture medium perfusion (Shih et al., 2013). Regardless of little adaptations, two liver-on-a-chip systems prevail: 2D- and 3D-based cell culture models (Ehrlich et al., 2019).

2D-based cell culture models are easier to manufacture and to maintain in cell culture, supporting early stage drug screening studies (Tilles et al., 2001). Banaeiyan et al. developed a microfluidic chip and cultured HepG2 and HLCs for mimicking the liver lobule microenvironment with a tissue-like hexagonal design and microchannels for convection-diffusion recapitulation of blood circulation (Banaeiyan et al., 2017). McCarty et al. created a two-input Christmas tree-like microfluidic device and cultured primary rat hepatocytes to simulate liver zonation (McCarty et al., 2016). The development of a collagen sandwich configuration microdevice by Hegde et al. resulted in well-connected hepatocytes and bile canaliculus formation over 2 weeks, while highly secreting albumin, collagen, and urea (Hegde et al., 2014).

As a result of improving hepatocyte phenotype and function, *in vitro* liver disease modeling is also refined by liver-on-a-chip platforms. Zhou et al. co-cultured hepatocytes and HSCs in a two chamber microfluidic device to model alcohol-induced fibrosis, detecting TGFb secretion through biosensing microsensors and ALD-resembling hepatocyte phenotypes (Zhou et al., 2015). Anti-steatotic drugs (e.g., metformin, pioglitazone) showed reduction of triglyceride and high-grade free fatty acids accumulation within hepatocytes cultured on chips (Lasli et al., 2019), mimicking *in vivo* testing. Kang et al. developed a sinusoid-on-a-chip cultivating PHHs and immortalized bovine aortic endothelial cells on a PDMS/PET microchanneled device to model hepatitis B virus (HBV) infection. HBV DNA was detected by PCR, revealing viral replication and indicating efficient hepatotropic infectious diseases modeling using microchips (Kang et al., 2017).

Although promising, 2D-based cell culture systems demonstrate significantly reduced hepatocyte function when shear rates surpass 0.5 dyn/cm^2^, while reducing perfusion flow limits oxygen delivery (Ehrlich et al., 2019). The protection of hepatocytes inside microfabricated grooves or microwells and the addition of oxygen carriers (e.g., emulsified fluorocarbon) to the cell culture medium can overcome the design limitations (Nahmias et al., 2006).

3D-based cell culture systems improve 2D models incorporating hepatic spheroids or LOs in alginate particles, polyvinyl resin or silica beads microdevices (Strain and Neuberger, 2002). Bavli et al. monitored the mitochondrial respiration of HepG2/C3A spheroids through oxygen-sensing beads co-encapsulated in collagen-layered microdevices, allowing the detection of minute mitochondrial activity shifts and dysfunctions (Bavli et al., 2016). Bhise et al. cultured HepG2/C3A spheroids in a GelMA-printed chamber for 30 days, acquiring hepatocytes with high liver-specific ALB, A1AT, ceruloplasmin, and transferrin secretion (Bhise et al., 2016). Similarly, Lee et al. developed a decellularized-liver ECM-derived 3D liver microchip which promoted an increase in hepatocyte *ALB* expression and CYP450 activity, improving acetaminophen metabolism (Lee et al., 2019). Zhu et al. applied perpendicular flow pressure to hepatocyte surfaces through a porous membrane, demonstrating cellular compaction and polarization (Zhu et al., 2016).

The development of liver spheroid- and organoid-chips is also a platform for personalized medicine studies and for liver injury mechanisms comprehension. Schepers et al. cultured primary and hiPSC-derived 3D organoids on a C-trap architectured liver-on-a-chip for 28 days, demonstrating the viability of patient-specific *in vitro* drug toxicity evaluation for personalized medicine goals (Schepers et al., 2016). Lee et al. designed a spheroid-based 3D model of ALD, co-culturing primary rat hepatocytes and stellate cells to mimic alcohol-induced liver tissue recovery. The spheroids demonstrated increased roughness when treated with 80 uL/mL of ethanol, while the reduction on hepatocyte viability and function indicated the recapitulation of reversible and irreversible ALD clinical conditions using liver-on-a-chip platforms (Lee et al., 2016).

## Discussion

The vast majority of liver modeling *in vitro* is still carried out by the use of static culture platforms (Panwar et al., 2021), resulting in the maintenance of metabolic competence for short periods. However, these models lead to accumulation of metabolites, lacking relevant tissue-tissue interfaces and spatial organization, key drivers of cell function. The application of physiologically relevant fluid flow and associated mechanical forces is also limited, impairing *in vitro* drug testing.

Alternatively, LOs and liver-on-a-chip technologies allow cellular microenvironment control more efficiently (Nakao et al., 2011), enhancing hepatocyte functions, resembling *in vivo* cellular responses to drugs, and better resembling liver physiology, representing an alternative to animal experimentation and predominant 2D models.

LOs recapitulate the liver metabolism better than other 3D models (e.g., matrix-embedded liver exosomes), being able to predict DILI and other toxicological effects. However, iPSC-derived organoids have an immature phenotype, presenting low CYP activity and ALB production (Cox et al., 2020). Additionally, organoids vary in size and batch, hampering standardization and challenging its applications in large scale drug testing (Thompson and Takebe, 2020). Furthermore, it is difficult to visualize dynamic changes in cell position, morphology, or function within these large, disorganized, and multicellular structures.

Liver-organ-chips can be used to evaluate phase I and II metabolism with higher accuracy and physiological integration, establishing complex and reliable cellular microenvironments, able to mimic liver-specific drug response (Prodanov et al., 2016). Compared to non-automated organoid culture, the liver-on-a-chip is more reproducible because of standardized protocols and bioengineering fabrication. Yet, due to high complexity and cost, liver-on-a-chip devices are not widely spread and unsuitable for high-throughput assays.

For future applications, the weighted selection or integration between LOs and liver-on-a-chip systems is vital for the targeted development of *in vitro* liver disease modeling and drug screening. Next generation models should incorporate critical cell types that are not organ specific, such as blood vessels, lymphatic vessels, nerves, and immune cells, better mimicking human physiology while driving an integrated human-on-a-chip elaboration through bioengineering techniques to overcome *in vivo* models dependency.
